# Discriminative Learning for Automatic Staging of Placental Maturity via Multi-layer Fisher Vector

**DOI:** 10.1038/srep12818

**Published:** 2015-07-31

**Authors:** Baiying Lei, Yuan Yao, Siping Chen, Shengli Li, Wanjun Li, Dong Ni, Tianfu Wang

**Affiliations:** 1 Department of Biomedical Engineering, School of Medicine, Shenzhen University, National-Regional Key Technology Engineering Laboratory for Medical Ultrasound, Guangdong Key Laboratory for Biomedical Measurements and Ultrasound Imaging, Nanhai Ave 3688, Shenzhen, Guangdong, 518060, P.R.China; 2 Department of Ultrasound, Affiliated Shenzhen Maternal and Child Healthcare, Hospital of Nanfang Medical University, Shenzhen, China

## Abstract

Currently, placental maturity is performed using subjective evaluation, which can be unreliable as it is highly dependent on the observations and experiences of clinicians. To address this problem, this paper proposes a method to automatically stage placenta maturity from B-mode ultrasound (US) images based on dense sampling and novel feature descriptors. Specifically, our proposed method first densely extracts features with a regular grid based on dense sampling instead of a few unreliable interest points. Followed by, these features are clustered using generative Gaussian mixture model (GMM) to obtain high order statistics of the features. The clustering representatives (i.e., cluster means) are encoded by Fisher vector (FV) for staging accuracy enhancement. Differing from the previous studies, a multi-layer FV is investigated to exploit the spatial information rather than the single layer FV. Experimental results show that the proposed method with the dense FV has achieved an area under the receiver of characteristics (AUC) of 96.77%, sensitivity and specificity of 98.04% and 93.75% for the placental maturity staging, respectively. Our experimental results also demonstrate that the dense feature outperforms the traditional sparse feature for placental maturity staging.

Over the past decade, ultrasound (US) imaging has been extensively applied in prenatal diagnosis and prognosis since it is radiation-free, direct-use, and low-cost^1^,^2^,^3^,^4^,^5^,^6^,^7^. B-mode US imaging is one of the most frequently used US imaging especially for placental maturity staging. Placental maturity evaluation is becoming one of the most frequently used functional evaluation of placental abnormalities such as fetal death, still birth, small gestational age, and various pregnancy complications^4^,^8^,^9^,^10^,^11^,^12^,^13^,^14^.

Placental function is an important index for direct assessment of fetal growth and development, and it can be used to ensure the fetus health by reflecting intrauterine growth conditions. However, subjective evaluation of calcification degree is highly dependent on visual observation, which may suffer from doctor’s misjudgment and discrepancies. Generally, subjective measurement requires training, experience, and knowledge of sonographers or radiologists to stage fetal placental maturity, which is quite challenging in relatively under-developed countries due to the lack of experienced sonographers or radiologists. In addition, tedious manual work causes fatigue, which might lead to the differences, variations, errors, and mistakes in the staging results. Also, placental staging based on B-mode gray-scale US images are related to calcification, image quality constraints, and other external conditions. To address the limitations of the current diagnosis and prognosis, a computer-assisted staging method using visual discriminative features is proposed in this paper. This method makes diagnosis and prognosis decision not only based on the doctor’s subjective diagnosis experience, but also decision scores from the developed learned models via training, which can obtain a more accurate US image interpretation for the placental function evaluation than the traditional methods^2^,^15^,^16^,^17^,^18^.

In the literature, automatic US placental maturity staging algorithms have been widely developed to reduce the incidence of judgment error, standardize medical tests, and reduce the doctor’s workload^4^,^8^,^10^,^11^,^13^,^14^,^15^,^16^,^17^,^19^,^20^,^21^. For example, in 1979, the first placental maturity staging method^14^ was proposed by Grannum *et al.* to divide the chorionic, substance, and basal plates of the placenta into four stages. However, this method relied on visual observation of placental US images to determine the calcification degree, which was highly dependent on the subjective judgment of the operator. Liu *et al.*^15^ proposed to automatically classify placental maturity using SVM classifier. A classification rate of 90% had been obtained based on the three quantitative parameters: gray variance, distortions, and kurtosis. However, this classification result is not accurate enough. At this moment of time, none of the existing methods have been applied for clinical practice, and developing a practical computer-assisted placental maturity staging method would be timely.

To develop a new staging algorithm for placental maturity evaluation, high accuracy for automatic assisting technology with proper image interpretation is essential. The conventional method first detects the points of interest, and then determines the invariant feature descriptors from these points. The widely used affine covariant region detectors include Harris^22^, Hessian, HarrisLaplace, and difference of Gaussian (DoG)^23^. The visual discriminative features from these regions are quite essential to provide fetal health interpretation and staging decision making for the sonographer^16^,^24^,^25^,^26^,^27^. Single feature often cannot detect all the fine details of the US image, and fusion of features outperforms single feature by providing more latent and intrinsic information^1^,^2^,^3^,^4^,^6^. The conventional and popular features in the literature include the local binary pattern (LBP), Haar feature, scale invariant Fourier transform (SIFT)^28^, and histogram of gradient (HoG). For example, in^29^, a US image retrieval method has been proposed based on SIFT, LBP, and image intensity value, which achieves quite promising results with publicly available dataset. Although there are numerous features and descriptors available for the classification task^3^,^19^,^25^,^30^,^31^,^32^,^33^,^34^,^35^, it remains a challenging task to find similar and relevant features for the placental staging task due to the complexity and negligible differences in the US image. In addition, placental US images are subjected to the complex illumination modification, exposure time change, and specular reflection by the imaging process, which further increases the difficulty of the staging problem. It is known that the widely used SIFT^23^ achieves superior performance compared to other descriptors such as color especially in the computer vision field. Meanwhile, local intensity order pattern (LIOP)^36^ and DAISY^37^ have been demonstrated as highly effective methods applied in the recognition and classification task due to their robustness to numerous variations and distortions. In view of this, the placental function evaluation is based on dense sampled visual discriminative features.

It is known that most extracted features from the original image space are redundant, indiscriminative, and in high dimension. Feature dimension reduction such as linear discriminant analysis (LDA), principal component analysis (PCA), locally linear embedding (LLE), and locality preserving projections (LPP) are traditional ways to project from a high dimensional subspace to a low dimensional subspace in a supervised or unsupervised way. LLE is one of the best dimension reduction method, however, this method does not consider the feature discriminability. Hence, it may be inapplicable and infeasible to be used in the placental function staging. Moreover, most traditional methods do not account for the generative models in discriminative learning. Unlike the traditional method, the extracted features are encoded into a histogram of occurrence by discriminative learning in order to further boost the staging performance. The most popular encoding methods are bag of visual words (BoVW)^38^, aggregated codes of BoVW extensions such as vector of locally aggregated descriptor (VLAD)^39^, and Fisher vector (FV)^23^,^40^. Compared with the conventional methods, FV is able to obtain high discriminative subspace with the complementary feature subspace exploration. In addition, a multi-layer FV method is proposed instead of single layer FV to incorporate spatial information to improve the staging performance. Since BoF framework is simple and effective, our study is based on the BoF framework using multi-layer FV.

Specifically, the BoF method builds a vocabulary based on various visual features such as SIFT, raw pixel intensity, LIOP, DAISY, and the combination of SIFT and intensity features. After clustering the visual features using the nearest matching in the vocabulary, a histogram in terms of the number of visual word occurrence is used to represent the respective images. To further boost staging performance, these visual features are divided into a distinctive group, and then the group representatives are identified by the clustering method. To the best of our knowledge, FV has never been applied for the placental maturity evaluation using US images, or in other imaging modality for maturity assessment. Since there is no uniform standard and successful application of automatic staging of placental maturity in the clinical practice, the 4-stages placental maturity^14^,^16^ algorithm based on placental variations, chorionic plate, placental substance, and basal layers is adopted. An automatic technique to stage the placenta maturity is developed according to thestandards specified in^14^ and gestational stages of placental chorionic plate. In our study, comprehensive experiments are conducted to verify the effectiveness of the gradient and high order statistics strategy for the quantitative assessment of placental maturity in the clinical application.

Apart from studying the potential for placental maturity staging using US imaging, our experiment shall illustrate the important role of affine invariant feature descriptor used in the FV approach plays in the placental maturity assessment. The main aim of this study is to reach a better understanding of the BoF mechanism and dense features for the placental function evaluation. This research is of vital importance in calcification progression and placental maturity diagnosis to establish the optimal placental evaluation. The development and use of such a tool could consistently increase diagnostic information used for the placental maturity intervention in various conditions. Overall, the study of US imaging for the placental maturity function evaluation has great significance and potential in numerous diagnostic applications.

## Experiment Results

### Experiment setup

**Figure 1:**
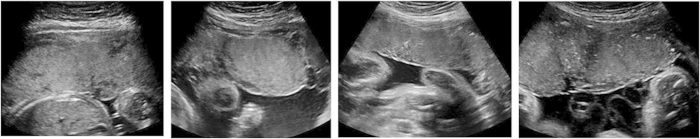
Image samples of 4 stages (from left to right, stage 0 to stage 3 image).

To assess the advantages and disadvantages of our proposed method, an extensive analysis had been conducted. The US images used in this study were acquired by a commercial US scanner (Acuson Sequoia 512, Siemens Medical Solutions, USA) from Shenzhen Maternal and Child Health Hospital. The representative placental images of each stage are shown in Fig. 1 (from left to right, stage 0 to stage 1). Our database is composed of a total of 443 placental images, where 187 images are in stage 0, 135 images are in stage 1, 85 images are in stage 2, and 36 images are in stage 3. The fetal gestational age in these placental images ranges from 18 to 40 weeks. Conventional US sweep was conducted to obtain images of pregnant women in the supine position by a radiologist having more than five years of experience in US obstetrics. In fact, the routine examination is performed by one radiologist at one time, but there are a total of three radiologists rather than one radiologist involved to provide the ground truth of the total 443 placental images in our study for placental staging, and 443 placental images are utilized in our study. Our system was implemented by the mixed programming technology using Matlab and C++. The feature extraction time for an image (size: 1024 × 768) is six seconds using a computer with a configuration of 32GBs RAM, double quad-core multi-threaded server with a single CPU, and the whole processing time for the testing step requires less than 1 second. The performance of the placental maturity staging is quantified by classification metrics such as area under receiver operation characteristics (AUC), sensitivity, specificity, and receiver operating characteristic (RoC) curves. The true positive rate is plotted on the x-axis while the false positive rate is plotted on the y-axis. All the experiments are 10-fold cross-validated to avoid any introduced bias. Every experiment is repeated at least 10 times and average results are reported in this paper. Quantitative assessment of the placental maturity of these four distinctive stages is evaluated in a BoF framework to evaluate the effect of different feature detectors, feature descriptors, feature encoding methods, and vocabulary sizes. A supervised learning method by the popular support vector machine (SVM) is selected to evaluate the staging performance since SVM method is capable of handling high-dimensional data and flexible enough to model diverse data sources. In addition, the original feature is partitioned into different subdivisions with multi-layer techniques, and histogram of all subdivisions are concatenated together to generate the final high level histogram feature vector. Without losing generality, this paper exploits a multiple-layer feature encoding method to make use of the spatial information.

### Effect of different feature detectors

**Figure 2:**
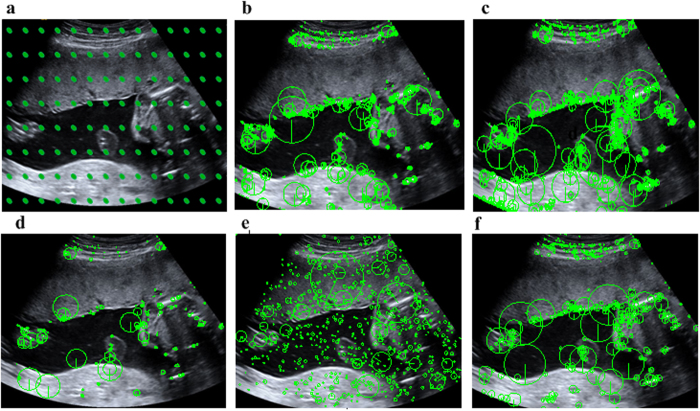
Different feature detector methods. (**a**) Dense sampling; (**b**) MultiscaleHarris; (**c**) MultiscaleHessian; (**d**) HarrisLaplace; (**e**) DoG; (**f**) Hessian.

**Figure 3:**
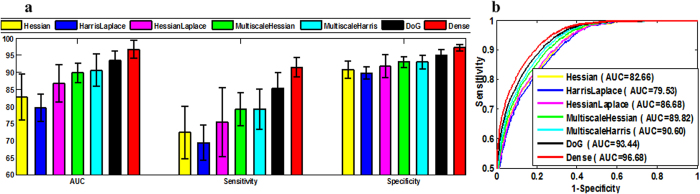
Staging results of different feature detectors in terms of Hessian, HarrisLaplace, HessianLaplace, MultiscaleHessian, MultiscaleHarris, DoG and dense sampling method. (**a**) mAP, sensitivity, specificity results of different feature detectors; (**b**) RoC curves of different feature detectors.

Figure 2 illustrates the dense sampling and popular interest point detector (IPD) methods such as HarrisLaplace, DoG, Hessian, MultiscaleHarris, and MultiscaleHessian. It is known that the brightness and calcification change information is quite essential and significant for the placental function staging. It is known that the detected points in the bright and edge parts are undesirable for placental staging. From the examples of the interest points captured in the US images, we observe the distinctive patterns for placental maturity evaluation. Figure 3a shows the mAP, sensitivity, and specificity results for four different stages and Fig. 3b shows RoC curve and AUC results of different feature detectors. It is observed that mAP is often higher than the sensitivity in the placental maturity staging. The DoG is quite desirable for this application since more interest points for placental evaluation can be detected from the placental image as compared to the selected IPD methods, but the dense sampling method is proved to be the most suitable and effective way for the staging evaluation. Compared to the traditional IPD methods such as MultiscaleHessian, Hessian, DoG, and MultiscaleHarris, dense sampling exhibits better performance than these traditional methods. The main reason is that these traditional methods may lose information during the feature extraction of placental images due to changes in brightness and calcification. The dense sampling describes everything in the placental images and is able to capture every region with calcification change, and hence best performance could be obtained with dense sampling as compared to the traditional IPD methods. This remarkable staging performance for the placental maturity evaluation indicates the effectiveness of the dense features.

### Effect of different feature descriptors

**Figure 4:**
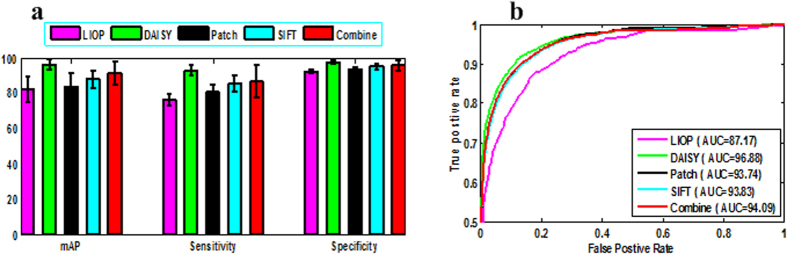
Staging results of different feature descriptors using dense sampling method. (**a**) mAP, sensitivity, specificity results of different feature descriptors; (**b**) RoC curves of different feature descriptors.

Figure 4 shows the staging performance of different feature descriptors in terms of mAP, sensitivity, and specificity based on the dense sampling method, where Combine denotes the feature combining SIFT and intensity information. It is noteworthy that DAISY feature outperforms the traditional SIFT feature in staging the placental maturity. Given the comparison result, the highest staging results are achieved with the DAISY feature. Although the combined feature of SIFT and intensity has produced good performance, it is still inferior to the DAISY feature descriptor. The preliminary explanation for the better staging performance is that DAISY feature not only has the same HoG feature, but it also adopts Gaussian weights and a circularly symmetrical kernel which permits staging performance to be superior to other traditional feature descriptor methods. Generally, the circular transform outperforms the conventional square descriptor, and the densely sampled features are suitable for the placental function evaluation and staging.

### Effect of different vocabulary sizes

**Figure 5:**
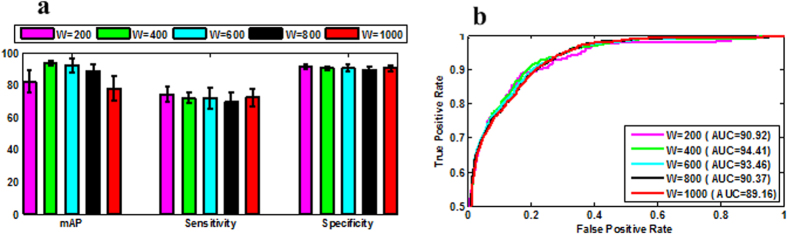
Staging results of SIFT descriptor using different vocabulary sizes in terms of W = 200, W = 400, W = 600, W = 800, and W = 1000. (**a**) mAP, sensitivity, specificity results of different vocabulary sizes; (**b**) RoC curves of different vocabulary sizes.

The BoF framework adopts a histogram of visual words from a vocabulary (a.k.a. codebook) for feature representation. This vocabulary is built using a GMM clustering algorithm by vector quantization in our experiment. The size of the vocabulary (i.e., W) is highly dependent on the image content and representations, and the vocabulary size is of vital significance in many applications 18,20 especially in the medical application. The effect of the vocabulary sizes on the staging performance is shown in Fig. 5. It can be seen that in most scenarios, a higher length of visual word yields better staging performance. In general, a smaller vocabulary size endows a higher discriminative power, and a higher vocabulary size has higher staging performance in most applications. However, an infinite vocabulary size does not guarantee the highest performance since the discriminative power can become saturated as size of vocabulary increases. A higher computation power is often needed for clustering, and an optimal trade-off between accuracy and efficacy can be obtained.

### Effect of different feature encoding methods

**Figure 6:**
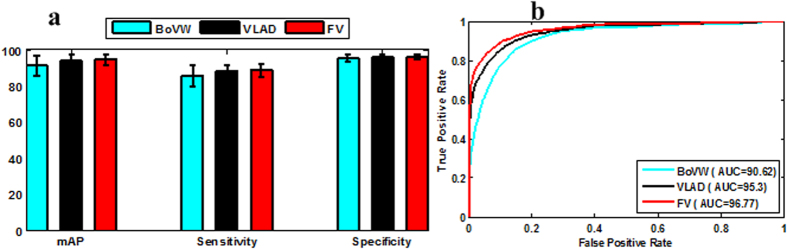
Staging results of feature encoding methods using SIFT descriptors. (**a**) mAP, sensitivity, specificity results of different feature encoding methods; (**b**) RoC curves of different feature encoding methods.

Figure 6a shows mAP, sensitivity, and specificity of the staging results, and Fig. 6b shows the AUC and RoC results obtained by various feature encoding methods based on DSIFT feature. It can be seen that the AUC result based on dense sampling with FV feature encoding method is 96.77%, and this value dropped to 95.3% and 90.62% with VLAD and BoVW feature encoding methods, respectively. It is clear that FV method outperforms the traditional VLAD and BoVW methods due to its higher statistics in the feature encoding method. Generally, aggregating vectors obtain better staging performance such as sensitivity, specificity, and mAP than the commonly used BoVW method. FV method obtains the best performance in terms of mAP, sensitivity, specificity, and AUC due to its discriminative learning and high order probability in the GMM model. It also indicates that the discriminative learning by FV and high order statistics are suitable and effective for the placental staging. Moreover, the obtained high staging result of the proposed method also demonstrates the potential practical application in the clinical practice.

## Discussions and Conclusion Remarks

The main goal of this work is to use the US data for placental maturity staging with a better understanding of the specific feature IPD and descriptor methods. For this work, the US image data is collected for placental maturity evaluation using BoF framework, and our extensive experiments prove that dense sampling and BoF framework are quite effective for the placental maturity evaluation. From the experimental results, it is observed that feature descriptor and detector are of vital significance for placental maturity evaluation. From our experimental results, it can also be seen that FV and dense feature are promising methods and have great potential in the placental maturity evaluation. From the experimental results, it is observed that DAISY descriptor achieves the best performance in all scenarios. From the effect of vocabulary size, it can be seen that W = 400 achieves a good trade-off between performance and efficacy in the placental evaluation. To the best of our knowledge, this is the first study on the application of BoF framework to evaluate placental maturity, and the overall experimental results are encouraging and suggest that BoF is a useful tool to evaluate the placental maturity.

**Figure 7:**
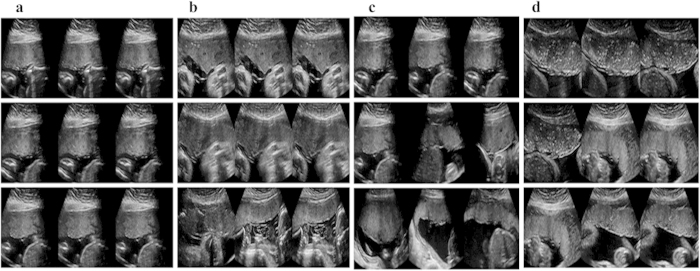
Query and top seven retrieved medical images. The first image is the query image, whereas the rest are the retrieved placental image based on the similarity scores (ranked from left to right, top to bottom). (**a**) Stage 1, (**b**) Stage 2, (**c**) Stage 3, (**d**) Stage 4.

To illustrate the effectiveness of the proposed method, the top seven similar images of the input placental images are plotted in Fig. 7. The first one is the input query placental US image, and the rest are the ranked placental US images based on the similarity scores (from left to right, top to bottom). Upon visual inspection, it can be seen that different stages can be accurately ranked. Based on the retrieved results using the similarity scores, it is also observed that placental images can be staged correctly when a new testing image comes from the retrieved results. It is obvious that the proposed method and extracted feature are highly effective for the placental maturity evaluation and staging.

To further compare our proposed algorithm with the related algorithm for placental maturity evaluation, Table 1 gives the detailed comparison results of our algorithm and the related algorithm, it can be seen that our proposed method obtains the promising results and outperforms the related algorithm. The main reason is that dense features have an advantage of extracting discriminative features, whereas the aggregating vectors are able to boost the staging performance by introducing high order statistics.

Overall, the automatic staging algorithm is an effective way to provide diagnosis assistance to evaluate the placental maturity evaluation. It will be useful in expediting the manual work of doctors and reduce time-consuming subjective measurement of clinician in clinical practice. The effectiveness of the proposed method is also validated by the achieved promising staging results. In fact, automatic staging in US imaging paves the way to reduce tedious work and training time for effective diagnosis. In addition, the methodologies utilized in this study are quite general and can be extended to classification and staging task in other fields.

In our future work, other advanced algorithms can be adopted to further enhance the staging performance. For instance, hierarchical fusion of the dense and sparse features is a suitable way to evaluate the placental maturity and placental function. In addition, other information such as the blood flow information is also beneficial in the placental evaluation. Fusion of classifiers should also be interesting in recognition of important placental stages. For example, unsupervised neural networks using deep learning can be incorporated in supervised SVM classifier to further improve its performance. Last but not least, segmentation and prediction algorithms can also be explored to boost placental maturity evaluation.

## Methods

### System overview

**Figure 8:**
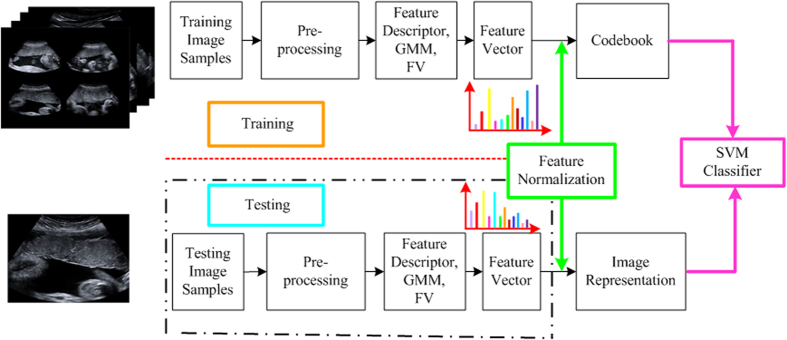
System overview of the automatic placental maturity staging method. The input images are first pre-processed (i.e., noise reduction), and then the features are extracted based on the dense sampling on the pre-processed placental images. FV is investigated to encode the extracted features and transform them to high-level representation in terms of histogram of occurrence.

**Figure 9:**
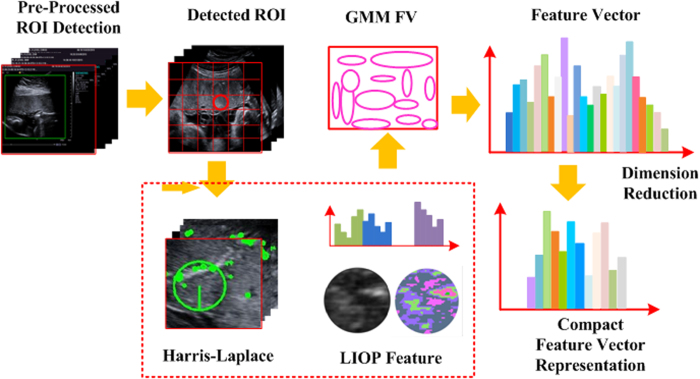
The procedure of feature vector formation. The input placental image is first partitioned into patches, and each patch is represented by the patch descriptor. GMM is applied to generate *k* Gaussians based on the assumption of a diagonal covariance matrix. The extracted feature is encoded by FV into histogram of occurrence as feature vector.

As illustrated in Fig. 8, an automatic staging system for placenta maturity is presented based on dense feature descriptor and FV. To further increase the discriminability of the descriptor and take advantage of spatial information, the original image is divided into various scales with multi-layer strategy. It is noted that spatial relationship among local appearances plays an essential role in recognizing the underlying structures in the US image. It is also proved that dense feature descriptor and FV feature encoding method obtains high descriptive power for the image representation. Also, spatial pyramid model achieves quite high staging performance compared with the traditional BoVW encoding method without spatial information. Therefore, spatial pyramid is also applied to divide the image into sub-divisions to incorporate this information. The densely sampled feature from each sub-division gains an advantage of single and individual region. The feature vectors are built by concatenating all features in every division and the original US image. Dense sampling is also applied to extract feature with a multi-resolution grid to fix the number of pixels between each sample. The means and variances of each visual word occurrence are concatenated to form the spatial layout of FV, which is the main difference between spatial pyramid model and spatial layout model. Figure 9 illustrates the feature vector formation by the proposed method in detail.

### Dense sampling

Traditional methods only obtain the gradient point in the distributed space to localize the interest point, and the fitting function is designed to find the fixed interest point, location and scale of the gradient point. Although the fitting function can reduce interest points of low contrast and unstable points to enhance the stability, the detected interest point in the placental images is still too sparse to be representative. The limitations of the IPD algorithm for the placental maturity staging are that the limited detected point is unable to describe the placental function quite well. Moreover, the placental images in different stages are with many variations. Hence, it is quite difficult to localize the interest region using the sparse descriptor. Also, the sparse descriptor has limited point and the detected point may have limited power to discriminate the small difference. Therefore, the accuracy is decreased due to indiscriminative features. By contrast, the dense descriptor extracts features from all the dense sampled points with a regular grid. Accordingly, dense sampling proposed in^41^ is utilized. In our method, both the neighboring and target regions are sampled using a slide window to extract the features. Dense sampling method can have better discriminability due to more discriminative points captured instead of the unreliable captured interest point by the IPD algorithms.

**Figure 10:**
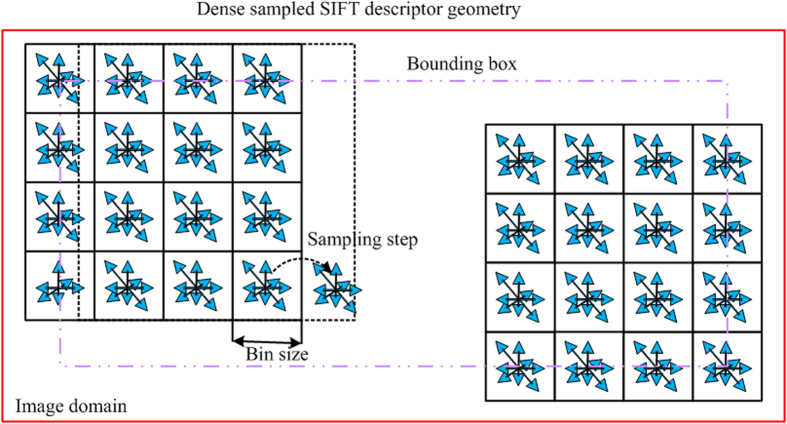
Illustration of dense sampling with SIFT descriptor.

Dense sampling, or grid sampling, is popular used for its simplicity and effectiveness. Dense sampling is performed on a regular grid, which causes a good coverage of the entire US image and a constant amount of features per image area. Figure 10 shows the dense sampling method with SIFT descriptor. Generally, regions with less contrast have less contribution to the overall image representation. The principle of dense sampling is that each patch has valuable information to represent the image content. Also, spatial relations with a regular pattern will help to interpret the US image. Namely, feature in a regular pattern will be easily interpreted in a simple model, and this spatial configuration of features is important to model the spatial relationships. Actually, dense sampling selects all the possible local features from the original US images by a fixed-size sliding window. If sampled by 1 pixel, it will lead to heavy computational cost. The sampling using sampling step helps a lot in reducing spatial complexity without losing much image information.

### Locally intensity order pattern

**Figure 11:**
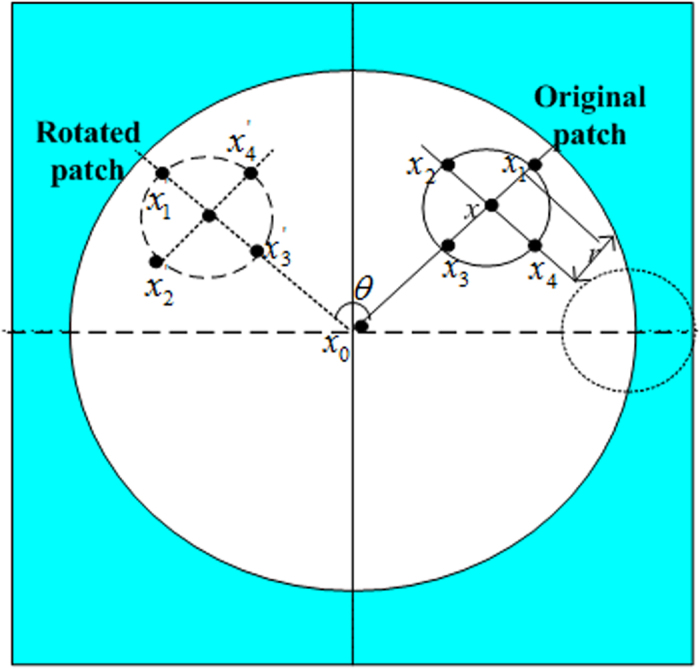
Layout of the LIOP descriptor, where shaded area is input patch (square area), white area is circular measurement region, and blue area is local neighborhood of a point.

LIOP is originally proposed to characterize local image luminance of order information^36^, and it is now widely applied as an important descriptor in the imaging field. LIOP is a local image descriptor obtained by sorting the selected image samples of increasing intensity using the local order pattern concept. The overall brightness of ROI is divided into a plurality of sequence information in each sub-region. This feature is invariant to light, monotonic intensity change of image, perspective changes, lossy compression, and image blur. The order patterns are rotation invariant^36^ by grouping the neighborhood sample around a pixel *x*, which is illustrated in Fig. 11. The points are anticlockwise sampled on a circle at a radius of *r*.

### Fisher vector

As defined in^42^, FV is a special case of the Fisher kernel construction. It is designed to encode local image features in a format that is suitable for learning and comparison with simple metric such as the Euclidean distance. Inspired by the promising performance of FV in^23^ for object recognition and classification task, FV has been investigated as a global feature encoding method to pool local image features to represent the placental image. Essentially, FV is derived from a special, approximated and improved case of the general Fisher kernel framework frequently. Inspired by remarkable results in^23^, GMM model is first implemented to obtain the posterior probability for staging performance enhancement. Specifically, a set of *D* dimensional feature vectors (e.g., SIFT descriptor) extracted from an image are fitted by a GMM model, and then the parameters in GMM model such as the mean and covariance are obtained. These parameters are incorporated in FV to increase the staging discriminability. Since the uncorrelated features and GMM covariance matrices of a diagonal assumption are consistent, PCA whitening is also applied to ensure that a diagonal covariance matrix assumption is satisfied.

## Additional Information

**How to cite this article**: Lei, B. *et al.* Discriminative Learning for Automatic Staging of Placental Maturity via Multi-layer Fisher Vector. *Sci. Rep.* **5**, 12818; doi: 10.1038/srep12818 (2015).

## Figures and Tables

**Table 1:** Algorithm comparison.

**Reference**	**Sample**	**Feature**	**Validation**	**Classifier**	**Result**
Linares^16^	59	textural features such as co-occurrence matrices, Laws masks and neighborhood gray-tone difference matrice	Leave one out	KNN	Best accuracy: 60.71%
Liu *et al.*^15^	200	gray level statistical feature: mean, variance, distortions, and kurtosis of gray scale.	120 training, 80 testing	SVM	Recognition rate 92%
Li *et al.*^20^	311	Dense sampling, DAISY descriptor.	Random partition	SVM	mAP: 92.5%, Sensitivity: 99.6%, Accuracy: 87.4%
Lei *et al.*^21^	443	HarrisLaplace, LIOP descriptor	Random partition	SVM	Accuracy: 93.75%,Sensitivity: 98.04% Specificity: 93.75%
Proposed	443	Visual descriptor such as DAISY, LIOP, SIFT, dense sampling	10 fold cross-validation	SVM	AUC: 96.77%, Sensitivity: 98.04%, Specificity: 93.75%
